# Emotional Distress Among Suicide Survivors: The Moderating Role of Self-Forgiveness 

**DOI:** 10.3389/fpsyt.2020.00341

**Published:** 2020-04-23

**Authors:** Yossi Levi-Belz, Tal Gilo

**Affiliations:** ^1^ Department of Behavioral Sciences, Ruppin Academic Center, Emek Hefer, Israel; ^2^ The Lior Tsfaty Center for Suicide and Mental Pain Studies, Ruppin Academic Center, Emek Hefer, Israel; ^3^ Department of Clinical Psychology, Ruppin Academic Center, Emek Hefer, Israel

**Keywords:** suicide-loss survivors, grief, bereavement, self-forgiveness, distress, depression, suicidal ideation

## Abstract

Grief after suicide entails unique challenges and difficulties, such as intense feelings of anger and guilt, as well as various psychological risks. The current study examined the contribution of self-forgiveness (SF) to emotional distress (e.g., depressed mood and suicidal ideation) among suicide-loss survivors, compared with bereavement following sudden and expected death types. Bereaved individuals (*N* = 309; aged 18-84) completed questionnaires measuring SF, depressed mood, suicidal ideation, demographics, and personal characteristics concerning the bereavement. A significant interaction between SF and type of loss was found, in which suicide-loss survivors with low levels of SF manifested the highest levels of depression and suicidal ideation compared with other subgroups. The findings reflect the importance of SF as a protective factor against depression and suicidality among suicide-loss survivors as well as the possible efficacy of forgiveness-based interventions in this population.

## Introduction

Suicide is one of the most disturbing public health problems. Each year, approximately 800,000 people worldwide die by suicide (1). Researchers estimate that every suicide has a direct and profound impact on roughly 60 people (2, 3), totaling between 48–500 million people who might be exposed to suicide bereavement each year (4). These people, termed *suicide-loss survivors*, are likely to be family members and spouses as well as friends and colleagues, who experience high emotional, physiological, or social distress during a lengthy period following the suicide of a significant other (5).

Accumulating evidence suggests that suicide bereavement involves unique challenges and difficulties compared with other types of bereavement. Suicide-loss survivors are more vulnerable to suffer from emotional distress and psychopathology, such as depression, PTSD, complicated grief (6–8), and even suicidal ideation and behavior (3). Moreover, suicide-loss survivors differ from other mourners in the thematic content of their grief (9–12). That is, suicide-loss survivors often face feelings of shame and stigmatization, leading to social withdrawal and concealment of the cause of death (10). Many of them are also haunted by agonizing questions, such as "Why did he do this to me?" "Did I do something wrong?" or "Could I have done something to prevent it?" (13). Thus, intense feelings of anger and guilt often become an integral part of the suicide bereavement process (9, 10). Given the magnitude of this complex struggle, it can be assumed that to make peace with past events and overcome their grief, suicide-loss survivors would be in need of certain mental resources.

Researches have indicated several personal and interpersonal characteristics that can ease the pain of suicide-loss survivors and help them to adaptively manage their grief. Recent findings have shown that self-disclosure, secure attachment, and perceived social support serve as protective factors against complicated grief (6, 14). Social support has also been shown to be associated with diminished loneliness and depression among suicide-loss survivors (15). Nevertheless, to date, the distinctions between suicide-loss survivors’ unique protective factors and those of other bereaved individuals have yet to be explored. A more comprehensive understanding of the internal mechanisms that may relieve distress, especially for suicide-loss survivors, is needed in order to improve clinicians’ ability to provide them with effective treatment adapted to their needs.

A personal characteristic that may play an important role in the management of distress among suicide-loss survivors is *self-forgiveness (SF)*. SF has been defined as a process in which one accepts his or her mistakes and wrongdoings, attempts to abandon anger or resentment toward oneself, and fosters positive emotions, thoughts, and behaviors toward the self (16).

The tendency to forgive oneself has been widely studied as a stable personality trait (17). It has been found to be related to various personal and interpersonal adaptive qualities, such as developed emotion regulation (18, 19), and high levels of positive relationships and social support (20, 21).

Accordingly, an extensive body of research has documented the positive contribution of SF to mental health, physical health, and well-being [(21–24); for an additional review, see (25)]. Specifically, SF has been found to be related to a decreased risk for depression and suicidal behavior among various populations (26–29). Furthermore, it has been shown that high SF serves to buffer emotional distress and psychopathological symptoms upon encountering stressors and life crises such as breast cancer (30) and traumatic events (31, 32).

Recent qualitative studies among suicide-loss survivors have shown that forgiving themselves is one of the toughest struggles in their bereavement process, yet, crucial for their recovery (33–35). Considering the strong guilt feelings and disproportionate responsibility that often characterize suicide-loss survivors, perhaps adopting some elements of SF could address their feelings and promote adaptive coping with them. To the best of our knowledge, however, no study has empirically examined the contribution of SF to emotional distress among suicide-loss survivors.

### The Present Study

In light of the importance of SF in dealing with traumatic and devastating events in general, and in line with the unique difficulties of suicide-loss survivors in terms of guilt and self-anger, in the present study, we aimed to examine the contribution of SF to emotional distress in the aftermath of suicide loss. In this study, suicide loss was compared with two other types of bereavement–following sudden death other than suicide and following expected death. We operationalized emotional distress as comprising depressed mood and suicide ideation, both reflective of the emotional disturbance of the individual. We suggest that SF's contribution to relieving distress among suicide-loss survivors will be greater than among the other two bereavement groups, considering the specific suicide-related bereavement characteristics (e.g., anger, guilt, and uncertainty regarding the possibility of having been able to prevent the death). Thus, we posit the following hypotheses:

H1: Suicide-loss survivors will report higher levels of depressed mood and suicidal ideation than will those enduring other types of bereavement.
H2: Suicide-loss survivors will report lower levels of self-forgiveness than will those enduring other types of bereavement.
H3: Higher levels of SF will be negatively related to levels of depressed mood and suicidal ideation in all bereavement groups but will have the most substantial contribution to diminishing depressed mood and suicidal ideation among suicide-loss survivors.

## Method

### Participants

The sample comprised 309 bereaved individuals (269 females [87.1%], 39 males), aged 18–84 (*M*_age_ = 44.3, *SD* = 16.23), divided into three groups:

**Suicide-Loss Survivors’ Group**: This group comprised 124 participants (108 females, 17 males, *M*_age_ = 40.22, *SD* = 13.80) who lost a loved person to suicide. Following Jordan and McIntosh (5), we defined *suicide-loss survivors* as individuals who lost a person close to them to suicide, and experienced emotional distress following the loss, regardless of their relation to the deceased. Accordingly, the participants were asked for their perceived level of closeness to the deceased, as well as for the level of distress experienced after the loss. We excluded participants who indicated no distress after the loss or absence of perceived closeness to the deceased.
**Sudden Death Group**: This group comprised 108 participants (98 females, 10 males, *M*_age_ = 53.97, *SD* = 17.06) who lost a person close to them due to a sudden death other than suicide (e.g., heart attack, car accident, terrorist attack, homicide, military combat).
**Expected Death Group**: This group comprised 77 participants (64 females, 12 males, *M*_age_ = 37.34, *SD* = 11.86), who lost a person close to them due to an expected death (e.g., old age or prolonged illness, such as cancer).

Exclusion criteria for the sample were being under age 18 at the time of the research and being under age 13 at the time of the loss. Informed consent was obtained from all participants.

#### General Demographic Characteristics of the Study Participants

The sample comprised 104 (33.7%) married participants, 92 (29.8%) widows, 82 (26.5%) singles, and 30 (9.7%) separated or divorced. Of the participants, 213 (68.9%) were parents. As for their religiosity, 220 (71.2%) identified themselves as secular Jews, 48 (15.5%) as traditional Jews, and 27 (8.7%) as orthodox Jews. Of the participants, 202 (65.4%) held a college degree, 50 (16.2%) had a secondary education, 54 (17.5%) had a high school education, and one participant had an elementary education.

#### Loss-Related Characteristics of the Study Participants

Kinship to the deceased consisted of 129 (41.7%) spouses, 77 (24.9%) children, 39 (12.3%) siblings, 19 (6.1%) parents, 17 (5.5%) friends, 10 (3.2%) grandchildren, and 10 (3.2%) other family members. The remaining nine (2.9%) participants identified themselves as acquaintances, colleagues, and ‘other’ kinship. Time since loss varied between 1 to 54 years (*M* = 16.59, *SD* = 13.66, Median = 15). The age of the participants at the time of the loss ranged between ages 13 and 75 (*M* = 29.61, *SD* = 11.61, Median = 26), and age of the deceased ranged between ages 14 and 94 (*M* = 39.84, *SD* = 16.4, Median = 39). Most of the sample reported suffering high (147, 47.6%) and severe (128, 41.4%) distress following the loss. Among the participants, 230 (74.4%) had sought psychological therapy after the loss, and 105 (34%) were still in treatment at the time of the study. Ninety (29.2%) participants had attended support groups following the loss, and 21 (7.8%) continued participating in them at the time of the study.

#### Group Differences in Demographic and Loss-Related Variables

Upon examining demographic and loss-related variables (see appendix A), several significant between-group differences were revealed: age (sudden death participants were older on average than the other two groups), time since loss (for the sudden death participants, more time had elapsed since loss), age of the participants at the time of the loss (suicide-loss survivor participants were older on average than the sudden death participants), age of deceased (the expected death participants reported older ages for the deceased), and perceived closeness to the deceased (the sudden death group reported higher levels of closeness than did the other two groups) as well as for family status, relation to the deceased and participation in support groups following the loss. Thus, these variables served as covariates in the data analysis. All other demographic and loss-related variables (e.g., gender, religiosity, psychological therapy after the loss and currently) yielded no between-group differences.

### Measures

#### Self-Forgiveness

SF was measured by the Heartland Forgiveness Scale [HFS; (36)]. The original questionnaire comprises 18 items, divided into three forgiveness subscales: Forgiveness of Self (Items 1–6), Forgiveness of Others, and Forgiveness of Situations. For the present study, we administered only the Forgiveness of Self subscale (e.g., “Although I feel bad at ﬁrst when I mess up, over time I can give myself some slack”; “I don't stop criticizing myself for negative things I've felt, thought, said, or done”). The items are presented on a seven-point Likert-type scale, ranging from 1 (*almost always false of me*) to 7 (*almost always true of me*). Higher scores reflect a greater inclination for SF. Following Thompson et al. (36), the subscale score was calculated by summing the items’ values. Cronbach's alpha reliability coefficient for this sample was α = 0.70.

#### Emotional Distress

Emotional distress was measured by two specific and direct questions:

1. *Depressed mood* was assessed by asking the participants for the frequency of feelings of depression, moodiness, or hopelessness during the past year. The item is scored on a five-point scale, ranging from 1 (*Never*) to 5 (*Very often* [*5 times or more*]).
2. *Suicidal ideation* was assessed using the Suicide Behaviors Questionnaire-revised [SBQ-R; (37)], measuring four dimensions of suicidality. For the present study, we used only Item 2 (“How often have you thought about killing yourself over the past year?”), which assesses the frequency of recent suicidal ideation. The item is scored on a five-point scale, ranging from 1 (*Never*) to 5 (*Very often* [*5 times or more]).*

#### Background Information

Background information collected from the participants included demographic characteristics (gender, age, education, family status, and religiosity) and loss-related characteristics (cause of death, time elapsed since loss, age of the deceased, age of the participant at the time of the loss, kinship to the deceased, perceived closeness to the deceased, levels of distress experienced following the loss, and use of health care services).

### Procedure

Recruitment of the participants transpired from September 2018 to January 2019, utilizing several platforms. Suicide-loss survivors were recruited primarily through the Facebook page of the nonprofit organization, *Path to Life*, the recognized organization for suicide-loss survivors in Israel. Participants of the other groups were recruited through Ministry of Defense organizations (*Yad LeBanim*, *IDF Widows*, and *IDF Orphans*), as well as through the snowball sampling technique (using online forums of bereavement and social media platforms).

All informants were provided a recruitment letter outlining the purpose of the study and the researchers’ contact information. The participants were assured of anonymity, confidentiality, and their right to withdraw from the study at any time. All participants completed the questionnaire online in a private setting.

### Data Analysis

A series of one-way ANOVA and chi-square analyses was conducted to determine group differences in demographic and loss-related variables. Then, a two-way MANCOVA analysis was conducted to examine the contribution of SF to emotional distress measures among different bereavement types, controlling for demographic and loss-related characteristics. An alpha of 0.05 was adopted for all tests of statistical significance. All analyses were conducted using IBM SPSS, version 20 for Windows.

## Results

### Group Differences in SF and Emotional Distress Measures

**Table 1** presents a comparison of the scores of the three groups on the levels of SF, depression, and suicidal ideation. Contrary to our hypothesis, no significant differences were found between the study groups in SF levels. However, as we expected, significant between-group differences were found in levels of emotional distress measures, as suicide-loss survivors reported higher levels of depression and suicidal ideation relative to the other two bereavement groups.

**Table 1:** A Group differences in demographic and loss-related variables (*N* = 309).

Type of loss					
Measure	1. Suicide-loss survivors (*n* = 124)	2. Sudden death (*n* = 108)	3. Anticipated death (*n* = 77)	*F / X^2^*	Post hoc (Scheffé, *p* < 0.05)
**Age**				*F* = 37***	1 = 3<2
*M* (*SD*)	40.22 (13.80)	53.97 (17.06)	37.34 (11.86)		
Range	18–71	20-84	18–70		
**Family status *N* (%)**				*X*² = 57.73***	
Single	39 (31.5%)	13 (12%)	30 (39.5%)		
Married	59 (47.6%)	30 (27.8%)	15 (9.7%)		
Separated/divorced	13 (10.5%)	13 (12%)	4 (4.2%)		
Widow	13 (10.5%)	52 (48.1%)	27 (16.9%)		
**Relation to the deceased *N* (%)**				*X*² = 175.42***	
Parent	26 (21%)	18 (16.7%)	33 (42.9%)		
Sibling	36 (29%)	1 (0.9%)	1 (1.3%)		
Child	15 (12.1%)	2 (1.9%)	2 (2.6%)		
Spouse	18 (14.5%)	82 (75.9%)	29 (37.7%)		
Grandparent	1 (0.8%)	0 (0%)	9 (11.7%)		
Other relative (e.g., aunt)	8 (6.5%)	1 (0.9%)	1 (1.3%)		
Friend	14 (11.3%)	2 (1.9%)	1 (1.3%)		
Acquaintance	0 (0%)	0 (0%)	1 (1.3%)		
Colleague	1 (0.8%)	0 (0%)	0 (0%)		
Other	5 (4%)	2 (1.9%)	0 (0%)		
**Years since loss**				*F* = 59.40***	1 = 3 < 2
*M* (*SD*)	11.36 (7.72)	26.43 (16.31)	11.20 (8.60)		
Range	1–34	1-54	1–46		
**Age of the participant at loss**				*F* = 4.35*	3 = 1,2; 1 > 2
*M* (*SD*)	31.81 (14.32)	27.38 (8.53)	29.22 (9.86)		
Range	13–69	13–75	14-48		
**Age of the deceased**				*F* = 35.74***	1 = 2 < 3
*M* (*SD*)	36.79 (15.90)	34.60 (11.02)	52.10 (17.43)		
Range	14–75	18–73	16-94		
**Perceived closeness to the deceased**				*F* = 10.58***	1 = 3 < 2
*M* (*SD*)	3.74 (0.58)	4 (0)	3.82 (0.45)		
Range	1–4	4–4	2-4		
**Participation in support group after loss *N* (%)**				*X*² = 6.0*	
Yes	36 (29%)	39 (36.1%)	15 (19.5%)		
No	87 (70.2%)	69 (63.9%)	62 (80.5%)		

All values given as M (SD); *p < 0.05, ***p < 0.001.

Additionally, to test the combined contribution of type of loss and SF to emotional distress levels, we conducted a two-way MANCOVA with time elapsed since loss, age of the participant at the time of loss, age of the deceased, and perceived closeness to the deceased serving as covariates due to their revealed between-group differences. In each MANCOVA, we examined one of the emotional distress measures as a dependent variable: depressed mood in the first and suicidal ideation in the second. To appropriately examine differences in SF levels, we recoded SF into three categories: low (Z < −0.75), medium (−0.75 < Z < 0.75), and high (Z > 0.75). We chose those Z scores to enable a focus on the contribution of very high levels and very low levels of SF.

### Depressed Mood

**Figure 1:**
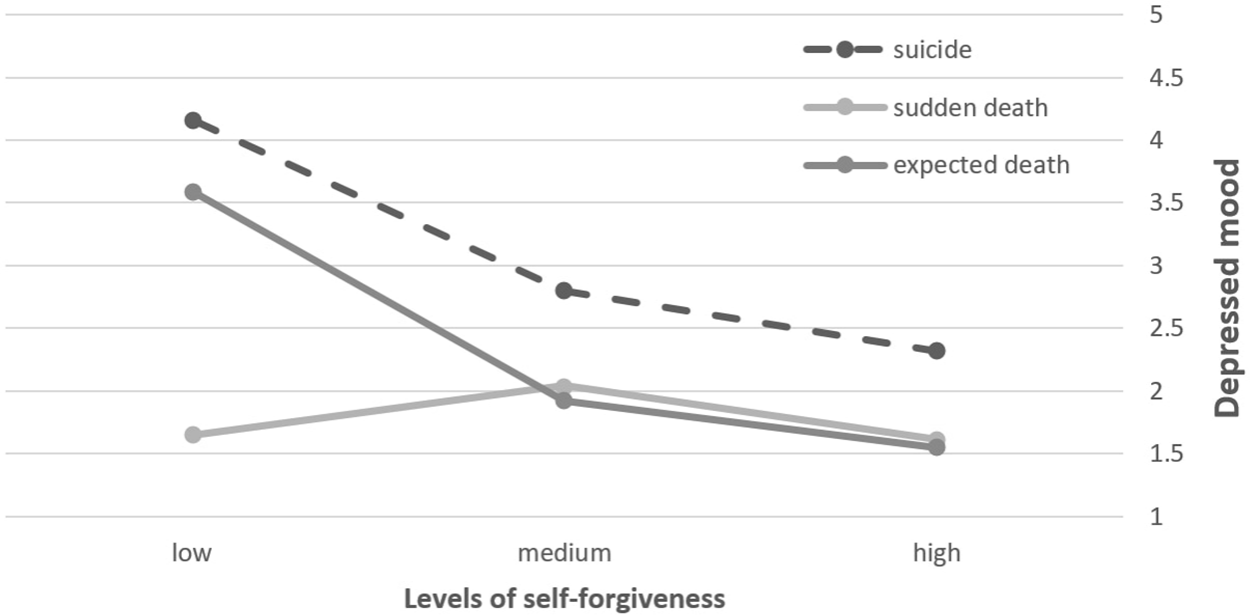
Depressed mood as a function of self-forgiveness (SF) and type of loss (*N* = 309).

As expected, we found a main effect of SF on depressed mood. As it can be seen on **Table 2**, Participants characterized by low SF reported higher levels of depression than did participants reporting the two higher levels of SF. More central to the hypothesis and evident in **Figure 1**, we also found a significant interaction between SF and type of loss on depressed mood levels. More specifically, suicide-loss survivors having low levels of SF reported the highest levels of depressed mood relative to all other groups and conditions. The differences in levels of depressed mood between low and high levels of SF were significant only in suicide-loss survivors, as well as in the expected death group. No significant differences between SF conditions in the sudden death group were found. Group differences in levels of depression were revealed only in the low and medium SF conditions, while the high SF condition was characterized by low depression levels, regardless of the type of loss.

**Table 2:** Means, standard deviations, and MANCOVA results of depression and suicidal ideation among the groups (N=309).

Dependent variable	Levels of Self-Forgiveness	Suicide survivors	Sudden death	Expected death	*F* Type of loss	*F* SF	*F* Type of loss * SF
		*n*=122	*n*=106	*n*=76	_(2,294)_	_(2,294)_	_(4,294)_
Depression	Low	4.16 (2.2)	1.58 (1.69)	3.59 (1.58)	6.54**	10.45***	2.92*
	Med	2.8 (1.87)	2.04 (1.63)	1.92 (1.53)			
	High	2.32 (2.13)	1.61 (1.74)	1.55 (1.63)			
Suicidal Ideation	Low	2.69 (1.54)	1.13 (.46)	1.76 (1.2)	21.79***	7.1**	3.44**
	Med	2.05 (1.25)	1.13 (.43)	1.35 (.79)			
	High	1.42 (.96)	1.22 (.60)	1.1 (.29)			

All values given as M (SD). *p<.05 **p <.01 ***p < 0.001. SF, Self-forgiveness.

### Suicidal Ideation

**Figure 2:**
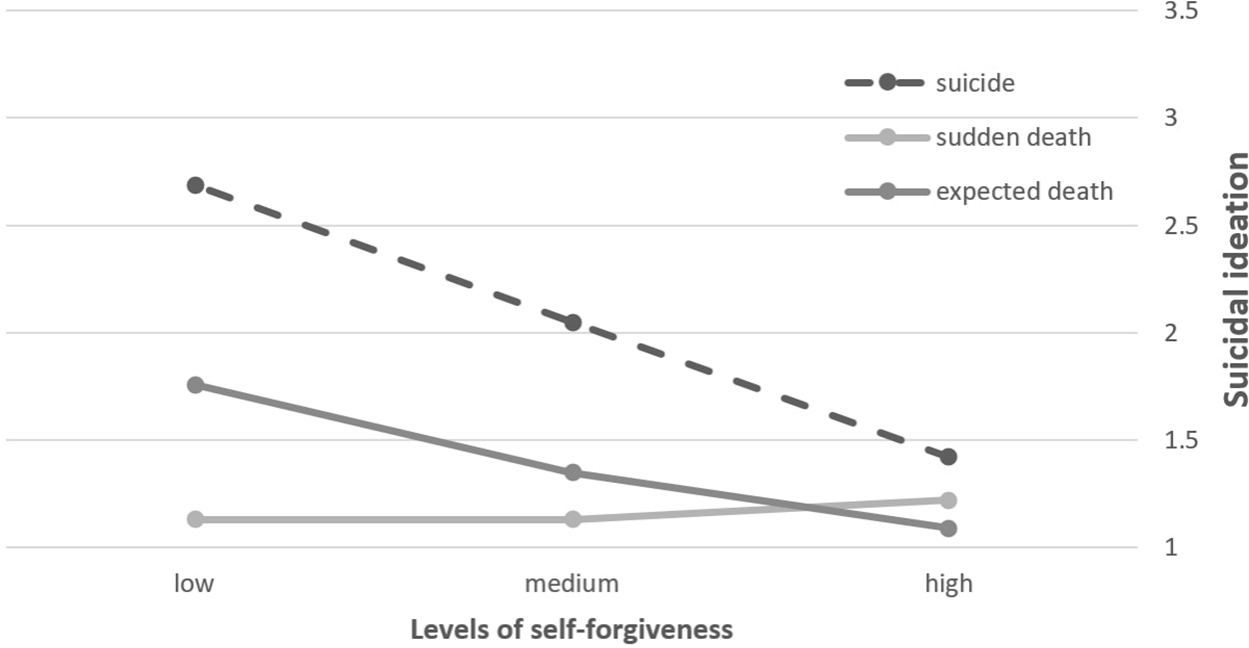
Suicidal ideation as a function of self-forgiveness (SF) and type of loss (*N* = 309).

We found a main effect for SF on suicidal ideation. As seen in **Table 2** and **Figure 2**, a significant interaction between SF and type of loss was found. The differences in levels of suicidal ideation between low and high levels of SF were the most prominent among participants in the suicide-loss survivors' group. Significant SF differences in suicidal ideation levels were also found in the expected death group, but to a lesser extent. Moreover, suicide-loss survivors with low levels of SF manifested the highest levels of suicidal ideation compared with all of the other subgroups, while suicide-loss survivors with high SF reported equal levels of suicidal ideation as other loss groups. Namely, consistent with our hypothesis, low SF comprises a risk factor for suicidal thoughts, particularly for suicide-loss survivors, whereas high SF buffers such thoughts.

In order to further probe the interaction effect, we conducted a moderation analysis using PROCESS software in SPSS. To pinpoint the difference in the relation between SF and suicidal ideation among suicide-loss survivors versus other types of grief, we recoded type of loss into two categories: suicide-loss survivors, and bereaved not by suicide. As seen in **Table 3**, among suicide-loss survivors there is a significant inverse correlation between SF and suicidal ideation. However, among those bereaved not by suicide, SF was found to be unrelated to suicidal ideation.

**Table 3:** Moderation analysis in predicting suicidal ideation among suicide-loss survivors in compare to other bereaved individuals (N=309).

Measure	*b*	*SE*	*95% CI*	*t*
Interaction SF * type of loss	−.28	.10	−.49, −.08	−2.76**
Suicide-loss survivors	−.37	.08	−.52, −.22	−4.91***
Bereaved not by suicide	−.09	.07	−.22, .05	−1.25

**p <.01 **p <.001 *** p< 0.001. SF, Self-forgiveness.

## Discussion

The primary goal of this study was to examine the role of SF as a moderator of emotional distress among suicide-loss survivors in comparison with two other bereavement groups––after a sudden death and after an expected death. Whereas a growing body of research has delineated unique challenges that characterize the bereavement process after a suicide (3, 9), the data regarding factors that may relieve distress in the face of their unique struggle are scant.

Overall, suicide-loss survivors in our study reported significantly higher levels of depression and suicidal ideation in comparison with the other bereavement groups. This finding is consistent with previous findings identifying an increased risk for a variety of psychological complications among suicide-loss survivors, especially mood disorders and suicidal behavior (3, 7, 38) as well as lower levels of posttraumatic growth (39). It is suggested that the high suicidal risk stems, among other sources, from the development of complicated grief, a common occurrence among suicide-loss survivors (40). However, other studies have revealed only minor differences concerning mental health outcomes between suicide-loss survivors and other bereaved individuals (for a review, see 12). Our findings may contribute to the resolution of this controversy by suggesting that suicide-loss survivors are indeed prone to more severe grief reactions, which may require targeted and sensitive care from professionals.

In line with our hypothesis, SF was negatively related to both depression and suicidal ideation measures. Previous studies have also highlighted the importance of SF as a facilitator of mental health and a protective factor against depression and suicidality (25, 27). Moreover, when we examined the combined contribution of SF and type of loss to depression and suicidal ideation levels, significant interactions were found. Namely, suicide-loss survivors with low SF reported the highest levels of both depression and suicidal ideation compared with all other subgroups. However, regardless of the type of loss, bereaved individuals with high SF levels reported relatively low depression and suicidal ideation levels. These findings highlight the role of SF as an internal resource that may buffer depression and suicidality among bereaved people in general and among suicide-loss survivors in particular.

Several possible explanations may be suggested for SF's role as a protective moderator for emotional distress among suicide-loss survivors. First, it has been shown that one of the pathways through which forgiveness promotes mental health is a decrease in rumination, stress, and negative emotions associated with unforgiveness, like guilt, shame, and regret (41, 42). *Intrusive rumination* (43) refers to repetitive, negative, and unwanted thoughts and relates positively to emotional distress during bereavement (44, 45). Bereaved individuals who are high in SF may be uninclined to engage excessively with their negative emotions, thus experience lower levels of distress. For individuals low in SF, however, the death circumstances may play a more prominent role in their ability to manage the bereavement process. That is, it can be suggested that among suicide-loss survivors, the combination of inevitably being occupied by questions such as ‘why’ and ‘what if,' along with unforgiving tendencies toward themselves, may exacerbate their distress and pain.

Another possible explanation for the protective role of SF lies in its relatedness to well-developed emotional skills, such as attending to feelings, identifying internal states, and repairing negative emotions (46). Such skills may help individuals high in SF to confront and work constructively with their emotional responses following negative experiences (47). Hence, it would appear that high-SF suicide-loss survivors could benefit from those skills in order to approach, digest, and manage painful feelings concerning their loved one’s suicide in adaptive ways, rather than being deterred or overwhelmed by them.

In light of the buffering effect of SF on suicide-loss survivors’ suicidal ideation, it is important to discuss the potentially inverse relationship between SF and self-aggression and harm. SF embodies the abandonment of aggressive, critical, and punishing dispositions toward the self in favor of positive ones, including compassion, caring, and even love (16). As such, SF is strongly related to elevation in health-promoting behaviors, such as seeking help or treatment (48). If so, this finding may reflect the importance of SF as an internal process allowing suicide-loss survivors to actively redirect energy toward healthy and constructive behavior and away from the destructive and health-impeding state of unforgiveness.

Several limitations of the current study should be noted. First, aiming to increase the sample size, we approached several organizations that target specific bereaved populations. Such groups are relatively homogeneous and thus may not fully represent all bereavement populations. Moreover, unique characteristics of those organizations' members might have produced between-group differences that were not taken into account in the current study. For instance, members of the Ministry of Defense organizations share not only similar sudden loss circumstances, but also specific cultural scripts related to death in the course of military service and terror attacks, as well as being granted governmental financial and psychological support. The fact that suicide-loss survivors do not receive such assistance can, to some degree, account for the group differences found in distress levels. A more representative sample of bereaved individuals from the community would enhance the generalizability of the study findings and limit the chances for intergroup variability.

Second, the use of self-report measures entails the risk of reporting bias. It is also necessary to consider the shortcomings of the measures used in our study. Since emotional distress was assessed by straightforward, individual questions, our findings need to be replicated using more objective distress measures. Third, the HFS assessed merely *trait* SF, which did not necessarily reflect the participants’ reactions to their specific loss (e.g., the suicide event), which might be influenced by personal and situational factors that were not evaluated. Future studies should examine both trait and state SF and compare their respective contributions to emotional distress among suicide-loss survivors.

Finally, the correlational and cross-sectional nature of this study precludes determining the sequence of the associations and inferring causality. Future research should consider a longitudinal methodology or a controlled examination of the effectiveness of a forgiveness-based intervention [e.g., (19, 49)] that could provide clearer indications as to the putative causal relationship between SF and distress among bereaved individuals. Moreover, in order to further deepen the knowledge regarding SF’s role in bereavement processes among suicide-loss survivors, it is important to examine the unique and specific contribution of SF to grief distress in a sample of suicide-loss survivors exclusively, compared with other well-established correlates of grief distress and difficulties. Our findings suggest that SF can serve as a protective factor against emotional distress in the aftermath of loss, especially among individuals bereaved by suicide. This study sheds light on the unique psychological risks associated with suicide grief and reveals the potential therapeutic influence of SF on suicide-loss survivors’ prospects of overcoming their pain and moving forward. Thus, the current findings suggest that interventions that promote SF, such as forgiveness therapy [FT; (49)], may be especially effective for suicide-loss survivors in managing their distress and fostering adaptive coping with their grief. This prospect is particularly invaluable, considering the lack of research demonstrating the efficacy of interventions for this population (2). From clinicians’ point of view, our findings suggest that identification of self-unforgiving tendencies in suicide-loss survivors during therapy may comprise a warning sign, alerting to their level of suicide risk.

## Data Availability Statement

The datasets generated for this study are available on request to the corresponding author.

## Ethics Statement

The studies involving human participants were reviewed and approved by Ruppin Academic Center Ethical Committe. The patients/participants provided their written informed consent to participate in this study.

## Author Contributions

YL-B and TG desiged the study, recruit the participants, conducted the analyses and wrote the final vertion of the manuscript.

## Funding

This work was supported by the American Foundation for Suicide Prevention (AFSP), under Grant [PRG-2-083-14].

## Conflict of Interest

The authors declare that the research was conducted in the absence of any commercial or financial relationships that could be construed as a potential conflict of interest.
